# Scientific impact factor versus social impact of journals

**DOI:** 10.5935/0004-2749.2024-1012

**Published:** 2024-10-08

**Authors:** Newton Kara-Junior

**Affiliations:** 1 Ophthalmology Department, Universidade de São Paulo, São Paulo, SP, Brazil; 2 Editor-in-Chief of Arquivos Brasileiros de Oftalmologia

Doctors publish scientific articles and other updates to become known as knowledge producers and be respected by their peers. Many doctors use different talents, being, at the same time, patients’ assistants, teachers, and researchers-an assemblage that generally gives them a position of leadership and prominence in the community.

The current challenge to be known as a researcher is that the main scientific journals have improved so much that it has become difficult for doctors not linked to universities or academic postgraduate programs to develop scientific research with adequate methodological rigor to be accepted and published. Methodological rigor is assumed as casuistic, follow-up time, approval by a research ethics committee, prospective study with a control group, and the relevance of the question to be answered by the study.

Even if it could be published, it may not produce the expected effect, as the community is no longer reading scientific journals as before when the printed issue arrived at their homes. Even if someone decides to read the issue online, they will not find it very interesting, as the information contained in articles of a high scientific level only makes sense when the background of that segment of knowledge is mastered, unlike when most articles published were easy to understand and by well-known authors. In high-impact science, each study adds a “very small brick to the wall of knowledge”, making random reading of articles boring.

In contrast, Brazilian researchers linked to universities and academic postgraduate programs are required to publish in journals with high scientific impact, which often leads them to publish their best studies in journals of American or English origin because the Internet has globalized science, bringing researchers and journals from all countries together^(1)^.

Likewise, Arquivos Brasileiros de Oftalmologia (ABO) receives good articles from many international and some national researchers. Faced with competition from international researchers, the selection process for accepted articles has increased, and there is less space left to publish science carried out by independent researchers without academic links or without a large support hospital, which allows them to perform research of high methodological level, which demands time, dedication, investment, and guidance.

The scientific quality of articles published in ABO has improved a lot in recent years, as has the journal’s impact factor, which is important to continue attracting good international articles and, little by little, the best articles from researchers linked to postgraduate programs. This creates a virtuous cycle in which quality attracts more quality, and national science wins.

However, much important information from in-de-pendent national researchers should be published. Spreading this knowledge through scientific channels is also a priority for us. Therefore, a training project in scientific research is being developed to train and guide young researchers on developing research with the appropriate methodological rigor to result in publication.

In ABO, sections with more democratic criteria for publication, such as the “letter to the editor” and “eye images”, were also created. However, to transcend scientific impact and effectively advance social impact, the most important action was the inauguration of the ABO page on social media.

The ABO page on Instagram (**@abojournal**) is a space to spread and discuss science without the pressure of bibliographic metrics but with the guarantee of academic rigor in publications. The editor comments on published scientific articles and didactically explains the context in which the evidence is included in the knowledge frame. National authors are invited to explain and interpret their articles published in ABO. Relevant articles sent by the community, photos, scientific comments, and other important elements are published.

The digital revolution in science provides alternative routes to spread discoveries, offers instant updates, and puts together editors and authors.

New-generation ophthalmologists have hunted notoriety through their posts on social media, which can be expensive, ethically questionable, and even arouse suspicion. Publications accepted on the ABO social media page are scientific and screened by editors and reach the ophthalmological community, enhancing the range and ensuring readers’ trust.

The scientific impact of journals is important for re-searchers, whereas social impact is important for the entire medical community. Thus, ABO wishes to com-pensate for its scientific specialization by approaching ophthalmologists through its social media and offering a new scientific dissemination channel.

The scientific impact factor is measured by the number of citations, from a few dozen authors to articles published in the journal, whereas the social impact factor is evaluated by the number of views and likes from hundreds or thousands of people interested in the subject. We want to be virtuous at both and, most importantly, remain close to our ophthalmological community.
